# TNFα Signaling Is Increased in Progressing Oral Potentially Malignant Disorders and Regulates Malignant Transformation in an Oral Carcinogenesis Model

**DOI:** 10.3389/fonc.2021.741013

**Published:** 2021-09-28

**Authors:** Jeffrey W. Chadwick, Rachel Macdonald, Aiman A. Ali, Michael Glogauer, Marco A. Magalhaes

**Affiliations:** ^1^ Faculty of Dentistry, University of Toronto, Toronto, ON, Canada; ^2^ Department of Dental Oncology and Maxillofacial Prosthetics, Princess Margaret Cancer Centre, University Health Network, Toronto, ON, Canada; ^3^ Department of Dentistry, Sunnybrook Health Sciences Centre, Toronto, ON, Canada

**Keywords:** carcinogenesis, squamous cell carcinoma, dysplasia, neutrophils, inflammation, TNFa

## Abstract

Oral carcinogenesis represents a multi-stage process which encompasses several genetic and molecular changes that promote the progression of oral potentially malignant disorders (OPMDs) to oral squamous cell carcinomas (OSCCs). A better understanding of critical pathways governing the progression of OMPDs to OSCCs is critical to improve oncologic outcomes in the future. Previous studies have identified an important role of tumor necrosis factor α (TNFα) and TNF receptor 1 (TNFR1) in the invasiveness of oral cancer cell lines. Here, we investigate the expression of TNFα and TNFR1 in human OPMDs that progress to OSCC compared to non-progressing OPMDs utilizing fluorescent immunohistochemistry (FIHC) to show increased TNFα/TNFR1 expression in progressing OPMDs. In order to interrogate the TNFα/TNFR1 signaling pathway, we utilized a 4-nitroquinoline 1-oxide (4-NQO) mouse model of oral carcinogenesis to demonstrate that TNFα/TNFR1 expression is upregulated in 4-NQO-induced OSCCs. TNFα neutralization decreased serum cytokines, inhibited the development of invasive lesions and reduced tumor-associated neutrophils *in vivo*. Combined, this data supports the role of TNFα in oral malignant transformation, suggesting that critical immunoregulatory events occur downstream of TNFR1 leading to malignant transformation. Our results advance the understanding of the mechanisms governing OSCC invasion and may serve as a basis for alternative diagnostic and therapeutic approaches to OPMDs and OSCC management.

## Introduction

Cancers affecting the oral cavity represent a heterogeneous group of disorders involving the region which is bounded posteriorly by the plane connecting the circumvallate papillae of the tongue and junction of the hard and soft palate and anteriorly by the mucosal surfaces of the labium superius and inferius oris. Approximately 75-90% of oral malignancies are oral squamous cell carcinomas (OSCCs) (1, 2). OSCC incidence varies widely depending on geographic region and can be influenced by primary etiological factors which commonly include tobacco, areca nut and alcohol exposure (3). OSCC diagnosis and staging are predicated upon clinical, radiographic and histopathologic evaluation with treatment assuming either single-modality or multi-modal therapy composed of a combination of surgery, external beam radiation and chemotherapy (4–7). Unfortunately, morbidity associated with treatment is considerable and long-term survival associated with advanced OSCC remains low (8, 9).

The development of OSCCs may be preceded by oral potentially malignant disorders (OPMDs) which represent a subset of conditions that possess an increased risk of progression to cancer (10). A 10-year review of the University of Toronto Oral Pathology Service showed that OPMDs are more prevalent than the combination of both benign and malignant tumors of the oral cavity (2). These conditions include, but are not limited to, leukoplakia, erythroplakia, lichen planus and oral submucous fibrosis. Risk assessment and management of OPMDs, despite unfettered access for direct visual examination of the oral cavity, is challenging. While several clinical parameters may increase suspicion of increased cancer risk, the mainstay for diagnosis and monitoring is tissue biopsy and histopathological evaluation to detect features of oral epithelial dysplasia (OED) or OSCC (11). There are many challenges in assessing risk of malignant progression in OPMDs. For example, the selection of biopsy location may not be representative of the entire lesion and histopathological grading of OED is often challenging and may not adequately stratify risk of progression. Other adjunct non-invasive diagnostic tests such as cytology, autofluorescence and spectroscopy have failed to consistently and reliably detect malignant progression (12–14). The need to identify molecular biomarkers capable of stratifying OSCC progression risk are in high demand. As such, unravelling the mechanisms by which OPMDs progress to OSCCs is critical for improving outcomes through the development of innovative diagnostic and treatment modalities.

The mechanisms governing malignant transformation are poorly understood which hamper our ability to provide interceptive therapy in the setting of OPMDs to reduce downstream disease morbidity and mortality. A dysregulated host immune system response within the tumor microenvironment has been identified as a significant factor influencing malignant progression (15, 16). Early work from our laboratory demonstrated a TNFα-dependent increase in OSCC invasiveness, invadopodia formation and matrix degradation (17). More recently, we also showed elevated neutrophil and lymphocyte in OPMDs and OSCCs as well as significantly elevated expression of TNFα in saliva samples of patients diagnosed with OSCC (18). Further, we characterized a phosphoinositide 3-kinase and Src-dependent mechanism for TNFα/TNF receptor 1 (TNFR1) mediated invadopodia formation and OSCC invasion *in vitro* but have not yet addressed this phenomenon *in vivo.*

Animal models of oral cancer have been developed and serve as indispensable tools for the study of the molecular and genetic mechanisms which govern oral carcinogenesis. These models are especially significant as the spontaneous occurrence of OSCCs in laboratory animals is exceptionally rare (19). Experimental induction of OSCCs within the mouse oral cavity through exposure to 4-nitroquinoline 1-oxide (4-NQO) is especially relevant for the study of both malignant and pre-malignant conditions. 4-NQO is a water-soluble quinoline derivative that facilitates DNA adduct formation and strand breaks (20, 21). Protracted administration of 4-NQO to mice via direct topical application or through its addition to drinking water produces a multitude of dysplastic, preneoplastic and neoplastic lesions which recapitulate the temporal, histologic and molecular progression of human OSCC development (22, 23). Other advantages of the use of this model over other popular xenograft and transgenic systems include the maintenance of an intact immune system and predictable progression to OSCC in an orthotopic manner (24, 25).

Here we utilize fluorescent immunohistochemistry (FIHC) and a semi-automated analysis algorithm on human samples of non-progressing and progressing OPMDs and demonstrate increased TNFα/TNFR1 signaling and inflammatory cell recruitment in progressing OPMDs. To determine the role of this pathway in carcinogenesis and progression, we utilize the mouse 4-NQO-induced oral carcinogenesis model and TNFα neutralization. The results show a decreased occurrence of invasive OSCCs in TNFα-neutralized animals. Using a similar FIHC approach, depletion of TNFα was found to reduce both TNFα and TNFR1 expression.

## Methods

### Human Study Population

A retrospective analysis was conducted utilizing oral biopsy specimens retrieved from the archives of the Toronto Oral Pathology Service (Faculty of Dentistry, University of Toronto) between December 2005 and January 2014. A total of 20 formalin fixed paraffin embedded (FFPE) samples were selected for analysis (**Supplemental Table 1**). The progressing cohort was comprised of 10 cases of OSCC (any subsite) possessing a previously submitted biopsy specimen demonstrating any grade of epithelial dysplasia from the same anatomic region of interest. The study cohort consisted of 10 progressing cases divided into three groups based on diagnosis: hyperkeratosis without dysplasia (HK; n=2), low-grade dysplasia (LGD; n=3) and high-grade dysplasia (HGD; n=5). The selected cases possessed a minimum of five years of histopathologic follow up, adequate material for analysis and no significant artifacts within the processed sections. The non-progressing cohort was comprised of 10 randomly selected cases of oral epithelial dysplasia which did not demonstrate histopathologic progression to OSCC over a period of nine years: HK (n=3), LGD (n=3) and HGD (n=4). All slides were reviewed by M.A.M. and A.A. The included cases of HK did not possess either the histopathologic or clinical criteria for a diagnosis of proliferative verrucous leukoplakia (PVL). The study was approved by the University of Toronto Research Ethics Board (Protocol 38933).

### Animals

A total of 43 six-week-old immunocompetent female C57BL/6 mice (Charles River Laboratories) were acquired and allowed a two-week acclimation period with *ad libitum* access to filtered water and standard chow in a temperature- and humidity-controlled environment with a 12-hour light and 12-hour dark cycle. All mouse studies complied with the relevant ethical regulations and were approved by the University of Toronto Animal Care Committee and the Research Ethics Board (Protocol 20011940).

### 4-NQO Administration

Animals were maintained on a normal chow. 4-NQO (Sigma) stock solution was prepared weekly in propylene glycol to a final concentration of 5 mg/mL and stored in a light-protected vessel at 4°C. The 4-NQO stock solution was diluted in the drinking water to a final concentration of 100 µg/mL and changed weekly in amber-colored bottles to prevent 4-NQO photodegradation. Mice were divided into an experimental group receiving 4-NQO-containing drinking water (n=33) or a control group (n=10) where drinking water contained propylene glycol (vehicle). Mice in both groups were allowed *ad libitum* access to drinking water. After a 16-week 4-NQO treatment period, mice from both groups were returned to and maintained on standard drinking water until sacrifice, independent of further experimental intervention.

### Antibody Administration

Following the 16-week treatment period, 13 mice that had received 4-NQO were randomly selected and divided into groups that received either 0.5 mg of InVivoPlus anti-mouse TNFα antibody (Clone XT3.11, BioXCell, n=6) or 0.5 mg of InVivoPlus rat IgG1 isotype control anti-horseradish peroxidase (HRP) (Clone HRPN, BioXCell, n=7). Both antibodies were diluted into InVivoPure pH 7.0 Dilution Buffer (BioXCell) and injected on a weekly basis for a total of eight weeks via intraperitoneal injection. Blood was collected via the saphenous vein for analysis prior to antibody treatment and at the four-week time point. All animals, including those that only received 4-NQO (n=20), were sacrificed at the eight-week time point. Those animals which did not survive until the experimental endpoint of eight-weeks (n=1, 4-NQO only; n=1, 4-NQO and anti-TNFα treatment) were excluded from the final analysis. Animals demonstrating oral lesions were euthanized prior to the experimental endpoint if lesions became ulcerated or resulted in deteriorating health conditions or pain as per standard operation procedures within the Division of Comparative Medicine including huddled posture, vocalization, hypothermia, or weight loss exceeding 20%. Blood and the entirety of the tongue were retrieved from all animals following humane euthanasia for analysis.

### Conventional Histopathology

Resected tongue specimens from experimental animals were immediately placed in 10% buffered formalin following their excision. Clinical photographs of the tongues were acquired prior to excision. Tissue samples were later bisected and one half was embedded in paraffin from which five-micron tissue sections were subsequently prepared and stained with hematoxylin and eosin (H&E). All slides were reviewed by M.A.M. and A.A for histopathologic characterization of oral tongue lesions using a DM2000 light microscope (Leica) at 100X total magnification.

### Fluorescent Immunohistochemistry (FIHC)

Five-micron sections were prepared from both human and animal formalin-fixed paraffin-embedded (FFPE) specimen blocks. After heating for 30 minutes at 60°C, slides were immersed in an antigen retrieval buffer (100X Citrate Buffer pH 6.0; Abcam) for one hour at 98°C followed by a wash with 1X Tris-buffered saline with 0.1% Tween^®^ 20 Detergent (TBS-T; Millipore) and permeabilized with 0.5% Triton X-100 (BioShop) for five minutes. The tissue sections were washed with TBS-T and then blocked in Sea Block Serum free -PBS (Abcam) for two hours at room temperature. Human sections were treated by overnight incubation with the following primary antibodies: Rabbit polyclonal anti-TNFR1 (1:200 dilution; Abcam), rabbit polyclonal anti-TNFα (1:250 dilution; Abcam), mouse monoclonal anti-CD45 (1:1000 dilution; Abcam). In the same manner, mouse sections were incubated overnight with the following primary antibodies: Rabbit polyclonal anti-TNFR1 (1:200 dilution; Abcam) and rabbit polyclonal anti-TNFα (1:250 dilution; Abcam). On the following day, slides were washed three times with 1X Tris-buffered saline and 0.1% Tween 20 detergent (TBS-T, Millipore) and incubated for one hour with the following secondary antibodies at room temperature: Anti-rabbit Alexa Fluor^®^ 586 (Abcam), anti-mouse Alexa Fluor^®^ 488 (Abcam). The tissue sections were then rinsed with TBS-T three times for five minutes and 4′,6-diamidino-2-phenylindole (DAPI; Thermo Fisher Scientific) was applied for 30 minutes. After washing with TBS-T three times for five minutes, slides were mounted with ProLong TM Diamond Antifade Mountant (Thermo Fisher Scientific) and imaged the same day. A second set of histology sections were stained using Alexa Fluor^®^ 594 anti-mouse Ly6G (1:500 dilution; BioLegend) which did not require secondary staining.

### FIHC Data Analysis

Ten images were acquired from multiple regions of each tissue section using the SP8 confocal microscope (Leica) for human samples and the Quorum Spinning Disk confocal microscope (Quorum Technologies Inc.) for mouse specimens. Data analysis was performed using Volocity Image Analysis Software (PerkinElmer) using a custom protocol that detected positive expression based on fluorescent intensity and area for both human and mouse specimens (26). Identification of morphological features within each tissue section was accomplished using DAPI. For each image, the region of interest (ROI) was manually defined to segment the epithelium and lamina propria (connective tissue) for human specimens and basal/parabasal epithelium and lamina propria for mouse specimens. Where present, the walls and the lumen of medium-sized blood vessels within the lamina propria were removed from the ROI. TNFα and TNFR1 positive cells were identified using an automated protocol based on pixel intensity, selecting those which were greater than or equal to three standard deviations (SD) above the mean intensity of the designated channel. Both the area of the positive pixels normalized to the area of the ROI as well as the mean fluorescence intensity (MFI) were calculated for each image and averaged from a minimum of five images per specimen. Those images with significant artefactual variations and background autofluorescence were removed from the analysis. Muscular layers and dilated blood vessels were cropped from each image before analysis. Neutrophil quantification from mouse specimens was accomplished by manually counting Ly6G^+^ cells in 10 high-power fields. The mean number of Ly6G^+^ cells per field was calculated and considered as the final neutrophil count for analysis.

### Flow Cytometry

Whole blood obtained via cardiac puncture at the time of animal sacrifice was fixed with fresh, methanol-free, 1.6% formaldehyde (Thermo Fisher Scientific) for 15 minutes on ice prior to processing. Red blood cell lysis was achieved with two sequential five minute treatments of 1x BD Pharm Lyse (BD Biosciences). Cells (5 x 10^5^) were resuspended in fluorescence-active cell shorting (FACS) buffer composed of 1x Hank’s balanced salt solution (HBSS) without calcium and magnesium (Gibco), 1% bovine serum albumin (BioShop) and 2mM EDTA (BioShop). Samples were blocked with 2 µg mouse (Sigma-Aldrich) and 60 µg rat (Sigma-Aldrich) serum for 20 minutes, labeled with anti-mouse Ly6G (1A8, BD Bioscience) and F4/80 (BM8, BioLegend) for 30 minutes on ice in the dark, and washed three times with FACS buffer. Sample acquisition was performed using the BD LSRFortessa X-20 flow cytometer with FACSDiva 8.0.1 (BD Biosciences) and analyzed with FlowJo v10.0.7 (Tree Star). Flow cytometer channel voltages were calibrated manually using Rainbow Calibration Particles (Spherotech) and compensation was performed with single-stained OneComp eBeads Compensation Beads (Invitrogen). Appropriate isotype control antibodies and fluorescence minus one (FMO) samples were prepared to establish negative staining characteristics for each antibody. A minimum of 2 x 10^5^ gated neutrophil events (Ly6G^+^F4/80^-^) were acquired for each sample.

### Cytokine Analysis

Serum was prepared from blood samples recovered from experimental animals at 0, 4 and 8 weeks following the termination of 4-NQO or vehicle exposure. Luminex^®^ assays were conducted in 96-well plates according to manufacturer instructions for the Millipore Mouse Cytokine/Chemokine Magnetic Bead Panel (MCYTOMAG-70K, EMD Millipore). Analytes included G-CSF, GM-CSF, M-CSF, IFN-γ, IL-1α, IL-1β, IL-2, IL-3, IL-4, IL-5, IL-6, IL-7, IL-9, IL-10, IL-12 (p40), IL-12 (p70), IL-13, IL-15, IL-17, LIF, LIX, IP-10, KC, MCP-1, MIP-1α, MIP-1β, MIP-2, MIG, TNFα, VEGF, RANTES and Eotaxin/CCL11. Briefly, plates were pre-washed and serum samples were incubated at 4°C overnight with buffer and mixed magnetic beads. Wells were then washed after which detection antibodies were added and allowed to incubate at room temperature for 60 minutes. Streptavidin-phycoerythrin was subsequently added to each well and incubated at room temperature for an additional 30 minutes. A final wash of the plate was then performed and followed by the addition of sheath fluid to each well. The plate was analyzed on the Luminex^®^ MAGPIX^®^ System at the Princess Margaret Genomics Center. The concentrations (pg/L) of cytokines and chemokines were quantified based on a standard curve and normalized to control samples.

### Statistical Analysis

One-way or two-way analysis of variance (ANOVA) and Tukey’s multiple comparison tests were conducted to assess differences between progressing and non-progressing OPMDs and Fisher's exact test was used to compare 4-NQO-induced tumor outcomes. All statistical analyses were performed using Prism 7.0 (GraphPad). The differences were considered statistically significant if p<0.05. Error bars represent standard error of the mean (SEM) unless otherwise indicated.

## Results

### Increased TNFα and TNFR1 Expression and Immune Cell Recruitment in Progressing OPMDs

**Figure 1:**
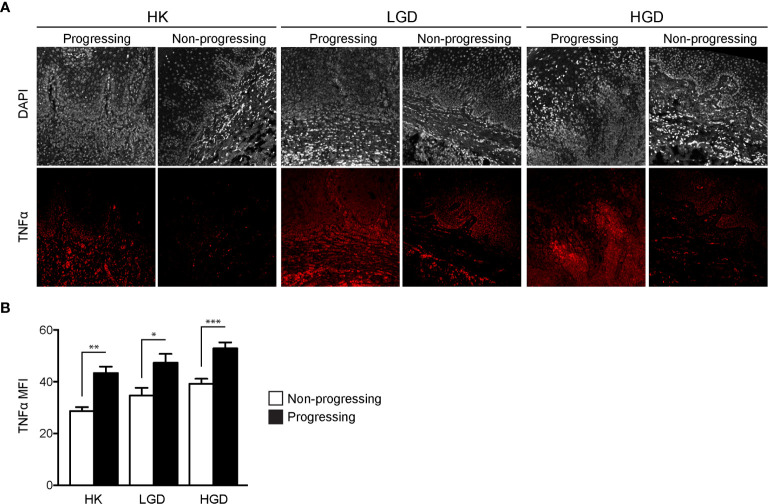
FIHC analysis of TNFα expression in human progressing and non-progressing OPMDs. **(A)** Representative FIHC images of human progressing and non-progressing patient OPMDs consisting of HK, LGD and HGD (white channel – DAPI; red channel – TNFα). **(B)** MFI of TNFα within the lamina propria of human progressing and non-progressing OPMDs. *p < 0.05; **p < 0.01; ***p < 0.001; HK, Hyperkeratosis; LGD, Low-grade dysplasia (mild dysplasia); HGD, High-grade dysplasia (moderate and severe dysplasia); MFI, Mean fluorescence intensity; DAPI, 4′,6-diamidino-2-phenylindole dihydrochloride; FIHC, Fluorescence immunohistochemistry; OPMD, Oral potentially malignant disorderdisorder; TNFα, Tumor necrosis factor α.

**Figure 2:**
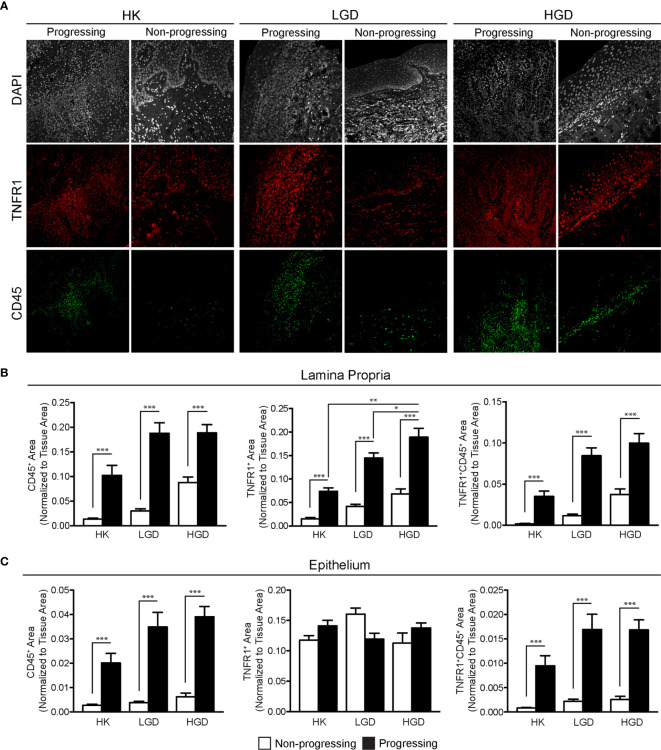
FIHC analysis of TNFR1 and CD45 expression in human progressing and non-progressing OPMDs. **(A)** Representative FIHC images of human progressing and non-progressing OPMDs consisting of HK, LGD and HGD (white channel – DAPI; red channel – TNFR1; green channel: CD45). **(B)** CD45^+^, TNFR1^+^ and CD45^+^TNFR1^+^ area within the lamina propria of human progressing and non-progressing OPMDs. **(C)** CD45^+^, TNFR1^+^ and CD45^+^/TNFR1^+^ area within the epithelium of human progressing and non-progressing OPMDs. *p < 0.05; **p < 0.01; ***p < 0.001; HK, Hyperkeratosis; LGD, Low-grade dysplasia (mild dysplasia); HGD, High-grade dysplasia (moderate and severe dysplasia; CD, Cluster of differentiation; TNFR1, Tumor necrosis factor receptor 1.

TNFα expression was quantified in oral biopsy samples using the MFI of TNFα within the lamina propria. A significant increase in TNFα MFI in progressing OPMDs as compared to non-progressing OPMDs (**Figures 1A, B**) was observed across all histologic diagnoses including progressing hyperkeratosis (p<0.01), progressing low-grade dysplasia (mild epithelial dysplasia, p<0.05) and progressing high-grade dysplasia (moderate and severe epithelial dysplasia, p<0.001). The expression of TNFR1 and OPMD-associated CD45^+^ immune cells (**Figure 2A**) within the lamina propria and epithelium of non-progressing and progressing lesions was also quantified as the average positive area per field. There was a significant increase in TNFR1 (p<0.0001) and CD45 (p<0.01) expression within the lamina propria of progressing samples compared to non-progressing samples (**Figure 2B**). Further, a significant increase in TNFR1^+^CD45^+^ immune cells (p<0.0001) was also noted in progressing lesions. While no differences were noted in TNFR1 expression within the epithelium, our results demonstrate an increase in intraepithelial CD45^+^ immune cells (p<0.0001) and TNFR1^+^CD45^+^ (p<0.0001) expression in progressing samples compared to non-progressing samples (**Figure 2C**).

### The 4-NQO Mouse Model of Head and Neck Cancer Produces an Array of OPMDs and Malignant Lesions

At the time of sacrifice, all animals that had received 4-NQO demonstrated mucosal abnormalities involving the tongue. Visual examination of the remainder of the gastrointestinal tract did not demonstrate gross disease in any of the subjects. Animals receiving only vehicle did not demonstrate any mucosal abnormalities along the entire GI tract. For those animals exposed to 4-NQO, tongue lesions were found to be both unifocal and multifocal and demonstrated a variety of gross appearances including hyperkeratotic papules, leukoplakic nodules and exophytic masses (**Supplemental Figure 1A**). It should be noted that regular examination under anesthesia following the cessation of 4-NQO exposure and prior to sacrifice was avoided due to the anticipated systemic fragility during carcinogenesis resulting in potential loss of subjects. Expectedly, animals treated with 4-NQO demonstrated significantly decreased body weight relative to controls following the completion of 4-NQO delivery and at the time of sacrifice (**Supplemental Figure 1B**). Microscopic evaluation of H&E stained tissue sections amongst those animals receiving 4-NQO revealed a relatively broad distribution of EH (epithelial hyperplasia), LGD, HGD, carcinoma *in situ* (CIS) and OSCC (**Supplemental Figure 1C**).

### Neutralization of TNFα Inhibits OSCC Invasion in Immunocompetent Mice

**Figure 3:**
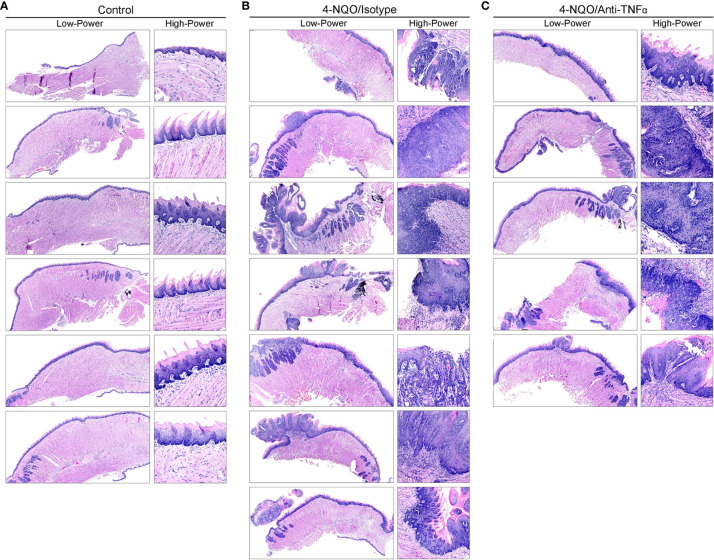
Mouse tongue resection photomicrographs. Representative H&E longitudinal sections (low-power: 10X; high-power: 20X or 60X) of mouse tongue specimens for **(A)** control (vehicle only, n=6) animals demonstrating normal tissue architecture, **(B)** 4-NQO/isotype antibody treated animals (n=7) demonstrating invasive features consistent with OSCC and **(C)** 4-NQO/anti-TNFα antibody treated animals (n=5) demonstrating only dysplastic changes. H&E, Hematoxylin and eosin; 4-NQO, 4-nitroquinoline 1-oxide; TNFα, Tumor necrosis factor α; OSCC, Oral squamous cell carcinoma.

To study the effect of TNFα blockade in oral carcinogenesis, immunocompetent mice received anti-TNFα antibody injections following the completion of a 16-week exposure to 4-NQO. As expected, control animals (n=6) that received only vehicle and no antibody treatment demonstrated a normal histologic architecture of the tongue (**Figure 3A**). Animals receiving 4-NQO and isotype antibody (n=7) developed lesions that were consistent with either OSCC (n=5, 71.4%) or dysplastic lesions suspicious for invasion (n=2, 28.6%) (**Figure 3B**). Animals receiving 4-NQO and anti-TNFα antibody therapy (n=5) showed dysplastic changes compared to vehicle-treated controls (**Figure 3C**). However, TNFα-neutralized animals did not develop invasive OSCC lesions, demonstrating only those histologic features consistent with high-grade dysplasia (p=0.0278, Fisher's exact test).

**Figure 4:**
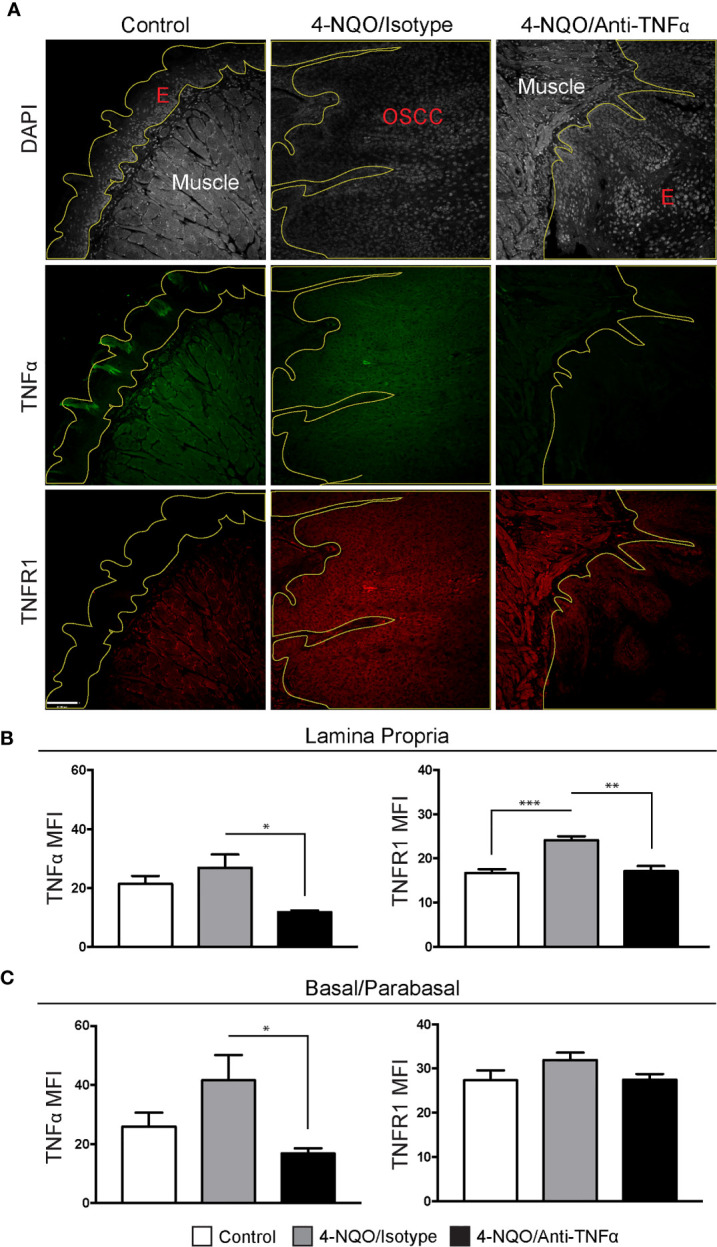
Neutrophil recruitment in the setting of 4-NQO-induced carcinogenesis. **(A)** Representative FIHC images of mouse tongue specimens from control (first column), 4-NQO/isotype antibody treated (second column) and 4-NQO/anti-TNFα antibdody treated (third column) animals (white channel – DAPI; red channel – TNFR1; green channel – TNFα; yellow line – demarcation of epithelium). TNFα and TNFR1 MFI within the **(B)** lamina propria and **(C)** basal/parabasal epithelium for control (n=6), 4-NQO/isotype antibody treated (n=7) and 4-NQO/anti-TNFα antibody treated (n=5) animals. *p < 0.05; **p < 0.001; ***p < 0.0001; E, Epithelium; MFI, Mean fluorescence intensity; 4-NQO, 4-nitroquinoline 1-oxide; FIHC, Fluorescence immunohistochemistry; MFI, Mean fluorescence intensity; DAPI, 4ʹ,6-diamidino-2-phenylindole dihydrochloride; TNFα, Tumor necrosis factor α; TNFR1, Tumor necrosis factor receptor 1.

To determine TNFα and TNFR1 expression, FIHC analysis was performed on specimens from vehicle, 4-NQO/isotype antibody and 4-NQO/anti-TNFα antibody treated animals (**Figure 4A**). TNFα and TNFR1 expression were quantified in an analogous manner to human samples. A significant decrease in TNFα MFI was noted in 4-NQO/anti-TNFα antibody treated animals compared to 4-NQO/isotype antibody treated animals within the lamina propria (**Figure 4B**) as well as the basal and parabasal layers of the epithelium (**Figure 4C**). There were significant increases in TNFR1 MFI within the lamina propria between control and 4-NQO/anti-TNFα animals relative to 4-NQO/isotype antibody treated animals (**Figure 4B**). No differences were noted in TNFR1 MFI within the epithelial layers between any group (**Figure 4C**).

### Chemically-Induced Carcinogenesis Induces Neutrophil Recruitment to Tissues and Neutrophilia in the Peripheral Circulation

**Figure 5:**
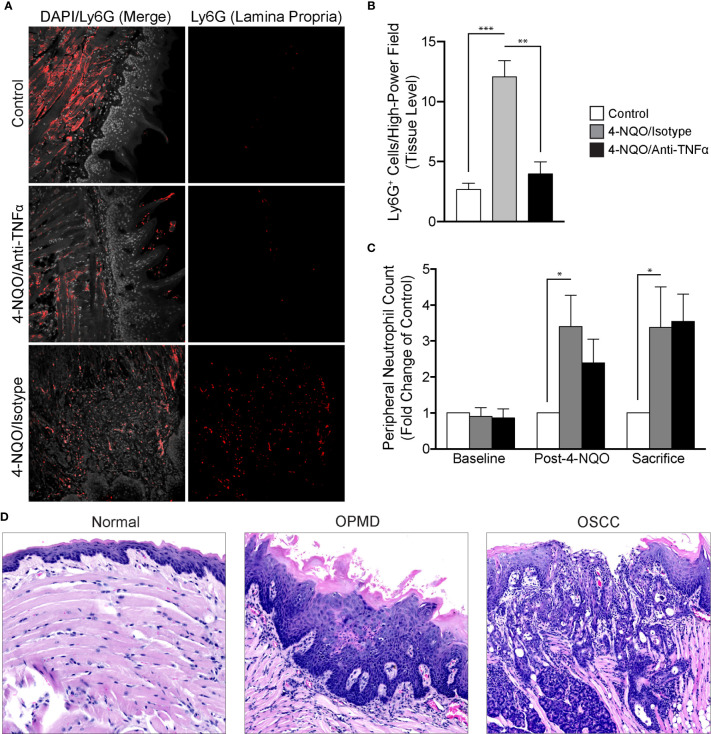
Analysis of neutrophil recruitment in the setting of 4-NQO-induced carcinogenesis. **(A)** Representative FIHC images of mouse tongues from control (first row), 4-NQO/isotype antibody treated (second row) and 4-NQO/anti-TNFα antibody treated (third row) animals (white channel – DAPI; red channel – Ly6G). **(B)** Quantitative analysis of Ly6G^+^ cells per high-power field for control (n=6), 4-NQO/isotype antibody treated (n=7) and 4-NQO/anti-TNFα antibody treated (n=5) animals at the level of the lamina propria. **(C)** Peripheral blood neutrophil count (Ly6G^+^ cells per 50 μL of blood) for control (n=6), 4-NQO/isotype antibody treated (n=7) and 4-NQO/anti-TNFα anditbody treated (n=5) animals. **(D)** Selected H&E longitudinal tissue sections demonstrating variations in inflammatory infiltrates between a histologically normal mouse tongue (left panel), mouse tongue afflicted with an OPMD (middle panel) and a mouse tongue demonstrating an OSCC (right panel). *p < 0.05; **p < 0.001; ***p < 0.0001; 4-NQO, 4-nitroquinoline 1-oxide; FIHC, Fluorescence immunohistochemistry; H&E, Hematoxylin and eosin; OSCC, Oral squamous cell carcinoma; OPMD, Oral potentially malignant disorder; DAPI, 4ʹ,6-diamidino-2-phenylindole dihydrochloride; TNFα, Tumor necrosis factor α.

To evaluate the recruitment of neutrophils in the setting of 4-NQO-induced carcinogenesis, we utilized FIHC to quantify Ly6G^+^ cells within the lamina propria (**Figure 5A**). Our results demonstrated a significant increase in Ly6G^+^ cells after 4-NQO treatment compared to controls (p<0.001). Neutralization of TNFα inhibited the recruitment of neutrophils to the tumor microenvironment induced by 4-NQO treatment (**Figure 5B**) with no significant differences between control and TNFα-neutralized animals (p>0.05). The increase in neutrophils in 4-NQO-treated animals at the tissue level was also reflected systemically as assessed by flow cytometric analysis of blood specimens collected at both the intermediate timepoint and sacrifice (**Figure 5C**). TNFα neutralization did not abate the systemic neutrophilia.

We also evaluated the inflammatory infiltrates in all treatment groups (**Figure 5D**). Increased severity as characterized by density of the infiltrate, extent as characterized by the depth of the inflammatory cell front relative to the epithelium, lamina propria and muscle as well as intraepithelial recruitment of the inflammatory infiltrate were assessed. There was an increase in the density, extent and presence of intraepithelial inflammation in animals treated with 4-NQO. TNFα neutralization decreased the presence of intraepithelial inflammatory cells. Expectedly, those animals not receiving 4-NQO showed minimal or a complete absence of any underlying inflammation. Further, the general variation in histologic diagnoses as well as inflammatory cell infiltration is reassuring in the use of this model to study malignant transformation as both OPMDs and OSCCs were observed amongst exposed animals.

### Cytokine Expression

Multiplex analysis demonstrated the differential expression of multiple cytokines between treatment groups (**Table 1**). Serum samples from animals within each group were combined for analysis at both the initial and four-week timepoints due to limited blood volumes collected by saphenous vein draws and analyzed individually at the time of sacrifice. There was a consistent increase in the concentration of cytokines in 4-NQO-treated animals that received isotype antibody treatment while the vehicle control and TNFα-neutralized animals showed minimal changes in the concentration of the cytokines over the duration of the experiment. At the time of sacrifice, there were significant increases in the concentrations of IL-1α, IL-12 (p40), IL-13, LIX, M-CSF, MIP-2 in 4-NQO/isotype antibody treated animals compared to non-4-NQO treated controls (**Supplemental Figures 2A, B**). These increases were inhibited by TNFα neutralization (**Supplemental Figure 2C**).

**Table 1:** Cytokine Expression.

Cytokine	4-NQO/Isotype			4-NQO/Anti-TNFα			Control		
	Post-4-NQO	Interim	Sacrifice	Post-4-NQO	Interim	Sacrifice	Post-4-NQO	Interim	Sacrifice
	Mean		Mean ± SEM	Mean		Mean ± SEM	Mean		Mean ± SEM
GM-CSF	177.00	91.00	168.00 ± 43.00	223.00	277.00	224.00 ± 58.00	151.00	2216.00	297.00 ± 52.00
Eotaxin	844.93	915.02	1299.87 ± 82.16	1029.99	841.25	1210.81 ± 139.31	972.31	974.90	1489.89 ± 63.40
G-CSF	9.68	202.61	240.60 ± 164.05	0.00	0.00	0.75 ± 0.745	0.00	0.00	0.00 ± 0.00
INF-γ	1.31	138.53	241.40 ± 160.10	0.49	0.00	3.94 ± 2.98	0.00	0.00	0.10 ± 0.10
IL-1α	90.07	2345.41	3672.72 ± 1366.41^a,b^	91.40	136.89	1028.73 ± 841.22^b^	20.39	38.58	15.95 ± 9.11^a^
IL-1β	6.34	5.52	3.59 ± 1.44	6.34	0.76	4.93 ± 2.26	1.72	0.02	0.00 ± 0.00
IL-2	16.23	202.74	330.06 ± 232.37	13.91	11.23	3.70 ± 2.26	10.13	3.61	1.11 ± 0.49
IL-4	1.17	1.38	1.36 ± 0.55	1.35	1.11	1.29 ± 0.54	0.94	0.46	0.58 ± 0.16
IL-3	0.00	0.00	1.32 ± 1.32	0.00	0.00	0.00 ± 0.00	0.00	0.00	0.00 ± 0.00
IL-5	11.02	5.20	3.20 ± 1.06	9.31	10.62	19.20 ± 9.82	14.41	13.38	8.79 ± 1.37
IL-6	4.58	2.70	14.35 ± 6.56	9.81	6.66	5.77 ± 1.59	0.00	7.66	1.57 ± 0.98
IL-7	4.64	14.29	5.34 ± 1.99	12.87	15.96	18.33 ± 9.22	0.00	0.86	36.21 ± 29.83
IL-9	15.10	403.72	1517.34 ± 1220.99	29.64	58.26	76.59 ± 28.39	32.21	7.95	25.41 ± 8.15
IL-10	1.71	8.19	16.22 ± 10.86	8.19	0.00	7.23 ± 4.90	2.33	3.67	2.10 ± 1.34
IL-12 (p40)	12.40	1356.41	2820.61 ± 1103.32^a^	14.83	30.15	724.02 ± 611.51	5.69	1.34	1.28 ± 0.61^a^
IL-12 (p70)	15.11	13.31	26.64 ± 14.91	18.89	1.19	5.74 ± 4.69	9.70	4.38	3.97 ± 2.43
LIF	0.58	1.10	0.87 ± 0.47	0.12	0.03	5.58 ± 3.91	0.03	0.95	20.03 ± 18.17
IL-13	136.70h	4331.21^d,e^	7817.62 ± 5577.97^a,b,c,f,g,h,i^	182.63^g^	105.04^c^	109.67 ± 10.23^b,d^	128.52^i^	110.65^f^	94.93 ± 9.35^a,e^
LIX	1208.43	2191.34	3169.96 ± 1058.76^a^	1147.59	745.36	1557.85 ± 643.45	817.99	1042.73	706.56 ± 416.86^a^
IL-15	24.43	67.93	74.38 ± 23.56	37.23	32.26	78.29 ± 42.94	24.40	92.96	1620.94 ± 1396.70
IL-17	6.94	11.24	10.85 ± 3.49	9.77	5.31	4.92 ± 1.31	2.36	2.95	0.87 ± 0.45
IP10	95.62	84.40	134.71 ± 11.66	103.67	83.76	160.07 ± 24.58	117.21	125.24	172.32 ± 7.09
KC	159.66	500.90	702.91 ± 418.18	198.15	106.48	160.54 ± 24.59	85.45	327.82	267.18 ± 74.71
MCP-1	38.21	34.61	33.99 ± 6.52	120.67	50.04	48.75 ± 10.49	36.43	60.10	40.52 ± 4.49
MIP-1α	72.04	490.44	519.76 ± 272.85	74.42	67.44	45.47 ± 15.42	49.12	29.49	13.14 ± 6.01
MIP-1β	16.04	184.85	242.62 ± 149.55	0.00	0.00	7.18 ± 3.49	4.37	0.00	0.00 ± 0.00
M-CSF	11.59	2001.69	3416.24 ± 1296.99^a,b^	10.08	38.22	788.99 ± 687.76^b^	5.97	3.74	1.99 ± 0.50^a^
MIP-2	104.22	2090.27	2636.20 ± 891.66^a^	104.10	246.02	877.20 ± 596.80	65.87	0.00	27.63 ± 11.68^a^
MIG	106.69	86.96	138.57 ± 26.04	683.41	89.54	187.39 ± 51.26	158.63	147.19	280.49 ± 24.93
RANTES	0.00	14.49	10.21 ± 3.30	10.03	0.00	6.66 ± 2.24	7.30	8.79	5.00 ± 2.69
VEGF	2.59	39.23	119.40 ± 100.54	1.81	1.38	1.34 ± 0.22	1.79	1.17	1.23 ± 0.22
TNFα	5.67	5.86	3.62 ± 1.58	6.53	1.86	4.95 ± 2.62	3.31	2.89	1.35 ± 0.38

Cytokine expression (pg/L) for control (n=6), 4-NQO/anti-TNFα antibody treated (n=5) and 4-NQO/isotype antibody treated (n=7) animals. Note 1: SEMs were omitted for post-4-NQO and interim timepoints as samples were pooled due to insufficient acquired blood volumes for each individual sample. Note 2: Post-4-NQO timepoint follows completion of 16 weeks of 4-NQO or vehicle delivery; Interim and sacrifice timepoints are four- and eight-weeks post-4-NQO delivery cessation, respectively. MFI, Mean florescence intensity; 4-NQO, 4-Nitroquinoline 1-oxide; SEM, Standard error of the mean; GM-CSF, Granulocyte-macrophage colony-stimulating factor; G-CSF, Granulocyte colony-stimulating factor; INF, Interferon; IL, Interleukin; LIF, Leukemia inhibitory factor; LIX, Lipopolysaccharide-induced CXC chemokine; IP, Interferon γ-induced protein; KC, Keratinocytes-derived chemokine; MCP, Monocyte chemoattractant protein; MIP, Macrophage Inflammatory Protein; M-CSF, Macrophage-colony stimulating factor; MIG, Monokine induced by interferon-γ; RANTES, Regulated on activation, normal T cell expressed and secreted; VEGF, Vascular endothelial growth factor; TNF, Tumor necrosis factor; ^a,b,c,d,e,f,g,h^p < 0.05; ^a^Control Sacrifice vs. 4-NQO/Isotype Sacrifice; ^b^4-NQO/Isotype Sacrifice vs. 4-NQO/Anti-TNFα Sacrifice; ^c^4-NQO/Anti-TNFα Interim vs. 4-NQO/Isotype Sacrifice; ^d^4-NQO/Isotype Interim vs. 4-NQO/Anti-TNFα Sacrifice; ^e^4-NQO/Isotype Interim vs. Control Sacrifice; ^f^Control Interim vs. 4-NQO/Isotype Sacrifice; ^g^4-NQO/Anti-TNFα Post-4-NQO vs. 4-NQO/Isotype Sacrifice; ^h^4-NQO/Isotype Post-4-NQO vs. 4-NQO/Isotype Sacrifice; ^i^Control Post-4-NQO vs. 4-NQO/Isotype Sacrifice.

## Discussion

Due to the poor survival and morbidity associated with late-stage OSCC diagnoses, prevention and early recognition of OPMDs is imperative. As the mechanisms of disease progression from an OPMD to OSCC are not fully understood, the efforts of our work have been focused at characterizing the immunoregulatory and inflammatory events governing this phenomenon.

**Figure 6:**
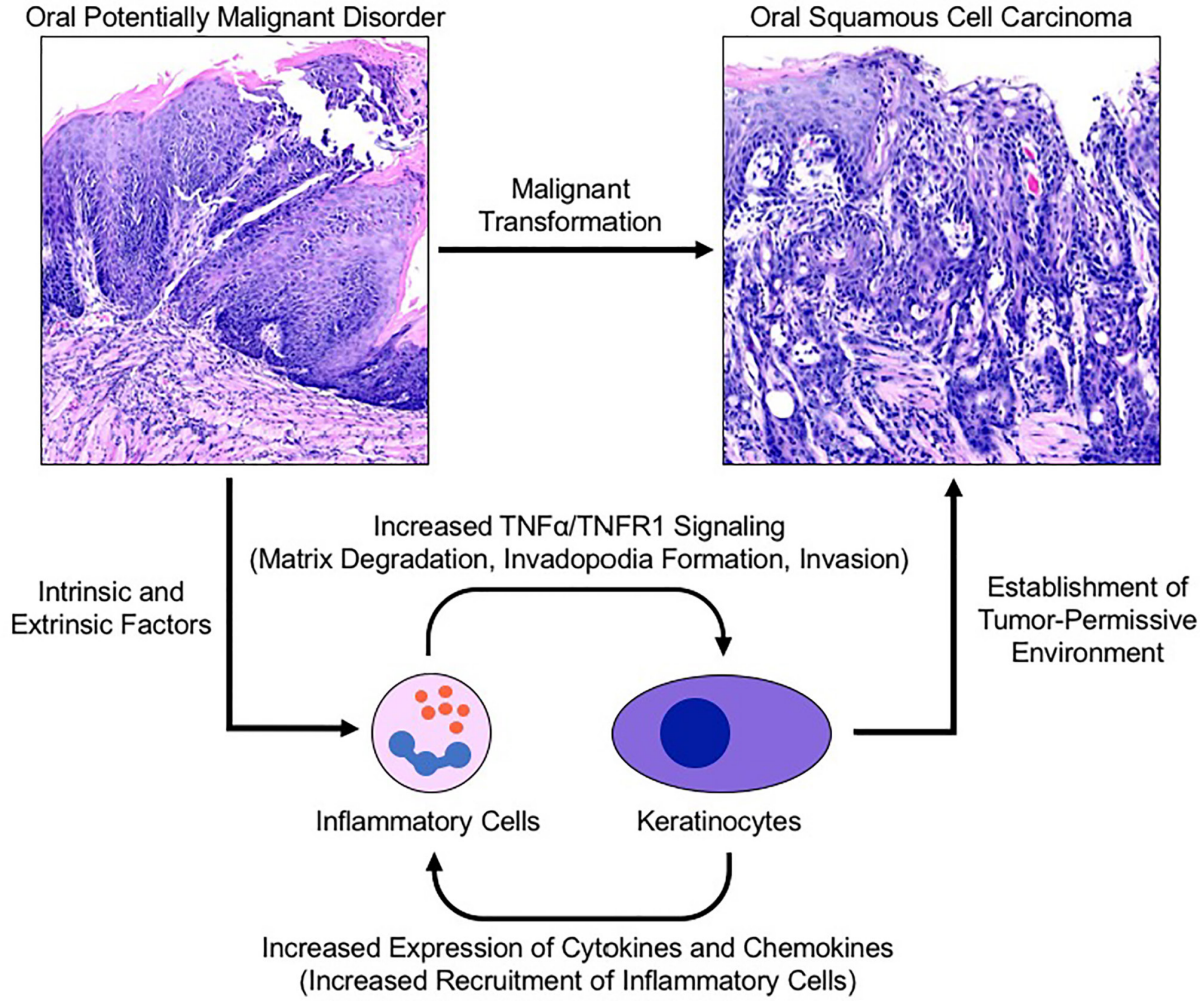
Proposed model for TNFα-induced malignant transformation in the setting of an oral potentially malignant disorder. Intrinsic and extrinsic factors induce oral premalignant disorders resulting in a cyclic signalling process between recruited neutrophils and keratinocytes to establish a tumor-permissive environment via the TNFα/TNFR1 signalling pathway. TNFR1 activation of keratinocytes enhances invadopodia development and matrix degradation thereby facilitating invasion. Pro-inflammatory cytokines released by keratinocytes recruit and activate neutrophils which assist with matrix remodelling and further activation of nearby keratinocytes. TNFR1, Tumor necrosis factor receptor 1; TNFα, Tumor necrosis factor α.

As the body of oncology literature evolves, both epidemiologic and clinical data continue to support the role of chronic inflammation in carcinogenesis. As a potent regulator of transcription and cell survival, TNFα has been implicated in the progression of multiple human cancers through promotion of tumor growth, angiogenesis, invasion and metastasis. The results from our human biopsy samples demonstrated a progressive increase in TNFα and TNFR1 expression as well as increased recruitment of CD45^+^ inflammatory cells from non-progressing OPMD samples to progressing OPMD samples, highlighting the crucial role of TNFα in the development of a pro-invasive environment. These results corroborate findings from our previous work which demonstrate that TNFα promotes tumor invasion and growth as well as expression of proinflammatory cytokines in an OSCC cell line (17). Further, TNFR1 knockdown has been shown to result in a decrease in oral cancer cell line invasion and inhibition of TNFα-induced invadopodia formation (18). These results in combination with our current findings showing increased TNFR1 expression in progressing OPMD samples is suggestive of a potential therapeutic target and is the first time that TNFα/TNFR1 signaling has been demonstrated to be elevated in progressing OPMDs. As such, we propose a model which suggests that these findings represent a novel mechanism linking oral inflammation and malignant transformation (**Figure 6**).

We studied the TNFα/TNFR1 signaling pathway using a 4-NQO-induced model of head and neck cancer utilizing immunocompetent C57BL/6 mice. While our group has utilized 3D *in vitro* invasion assays for the study of OSCCs, this model allowed us to evaluate these pathways in an immunocompetent animal model. Our results support the continued use of this model as a wide distribution of dysplastic as well as invasive lesions can be achieved as noted at the time of sacrifice. At this time, however, little is known about the inflammatory response as a result of the development of 4-NQO-induced lesions. Our results support an integral role of tumor-associated inflammation in oral carcinogenesis. As such, the use of ideal models of disease for *in vivo* study cannot be overstated. The application of the 4-NQO-induced model of oral cancer recapitulates dysplastic progression leading to the development of OSCCs in a similar manner to the human condition whilst maintaining an intact immune system. The latter point, in this case, is of critical importance as chronic inflammation associated with malignant progression plays a significant role in disease progression. Further, in the context of tumor evaluation following 4-NQO exposure, it is crucial to report all histopathologic features, as were noted in this study, and ensure that a representative number of sections have been evaluated as OPMDs and OSCCs are not homogenous entities.

Anti-TNFα antibody therapy has been demonstrated to exhibit robust anti-tumor effects in mouse models of pancreatic cancer and achieve disease stabilization in individuals with diagnoses of metastatic breast and recurrent ovarian cancer (27–29). Here, we demonstrate that anti-TNFα antibody treatment following the administration of 4-NQO attenuates or retards malignant transformation during a period where an untreated animal cohort went on to develop invasive lesions. Further studies are needed to determine whether the dysplastic lesions from anti-TNFα antibody treated animals would eventually progress to OSCCs, albeit at a slower pace, or if progression is completely inhibited. Our current findings are suggestive of a potentially critical role for the TNFα/TNFR1 signaling pathway in the context of carcinogenesis and malignant progression through the inhibition of tumor invasion and modulation of the inflammatory response to malignant processes.

We show the differential expression of a number of cytokines in the context of 4-NQO-induced carcinogenesis and TNFα blockade. Previous work from our group has shown a relative increase in salivary expression of proinflammatory cytokines including IL-1β, IL-6 and TNFα in patients with a diagnosis of OSCC compared to control patients with IL-1β and IL-6 demonstrating especially increased expression in advanced disease. In the context of 4-NQO-induced oral cancer, attention should be drawn to several findings at the time of animal sacrifice with respect to cytokine expression. First, there was a reduction in TNFα antibody concentration during anti-TNFα antibody administration as compared to isotype antibody treated controls which was not seen at the time of sacrifice. This suggests that in early stages of carcinogenesis, the aforementioned dosing of anti-TNFα antibody attenuates TNFα expression during treatment but becomes inadequate at later timepoints. While there were no invasive lesions noted on histologic analysis of specimens from the anti-TNFα antibody treated group at the time of sacrifice, it is plausible that with additional time, these lesions may have undergone malignant transformation or, alternatively, new foci of disease may have manifested. Even in the setting of mice deficient in TNFα, carcinogen-induced skin lesions will eventually develop in a significantly delayed fashion relative to control animals of multiple genetic backgrounds (30). Second, as compared to the intermediate time point, there was a significant upregulation of several cytokines which, with the exception of M-CSF, were not significantly different between the isotype and anti-TNFα antibody groups. This suggests that despite persistent TNFα blockade, cytokine expression was normalized to that of those animals treated with isotype antibody. As such, to ascertain the true effect of anti-TNFα antibody treatment in the setting of 4-NQO carcinogenesis, a thorough evaluation of expression differences at the intermediate timepoint was required which revealed several differences between the groups. As the initial and interim serum cytokine analyses used a pooled sample from all animals within each group, statistical analysis could not be performed at these time points. Despite this limitation, many clear differences could be seen from the dataset with the 4-NQO/isotype antibody treated group showing increases in concentrations of most cytokines including IL-1α, IL-12 (p40), IL-13, LIX, M-CSF, and MIP-2 while IL-1α, IL-12 (p40), IL-13 and M-CSF were significantly reduced in the setting of TNFα blockade. The aforementioned cytokines are all known to participate in immunoregulatory processes of both the innate and adaptive immune system in the setting of malignant processes (31–33). Similarly, the elevated chemokines M-CSF, MIP-2 LIX, have been implicated in specific mechanisms of invasion and metastasis (34–36).

Neutrophils are avid participants in carcinogenesis and metastasis and play an integral role in cancer progression by adopting both tumor-promoting and immunosuppressive functions which facilitate angiogenesis, invasion, migration and metastasis (37, 38). With respect to circulating neutrophils, while variations exist in the degree of association, an elevated neutrophil-to-lymphocyte ratio (NLR) has repeatedly been associated with poor survival outcomes in the context of numerous malignancies (39). In the setting of OSCCs, the NLR has demonstrated some utility as a predictor of survival (40). At the tissue level, it has been suggested that high levels of intratumoral neutrophils are associated with both decreased overall survival as well as diminished periods of recurrence-free, disease free and cancer-specific survival (41). We observed neutrophilia in both isotype and anti-TNFα antibody treatment groups at both the four- and eight-week time points relative to controls. While our results grossly recapitulate the neutrophilia noted with human cancers, there was a decrease in circulating neutrophils at the four-week time-point between the isotype and anti-TNFα treated groups, albeit not statistically significant. While this was likely attributed to the concurrent anti-TNFα antibody administration which was eventually overcome through continued and progressive neutrophil influx up to the point of sacrifice, this finding highlights a potential mechanism by which the inflammatory response significantly alters cancer progression. Recent work from our laboratory has also explored neutrophil influx within the oral cavity in the setting of OSCCs and demonstrated significantly elevated numbers of oral neutrophils relative to control subjects based on CD45^+^CD66^+^ cell counts by flow cytometry. The administration of anti-TNFα antibodies may exploit key immunomodulatory pathways which block or attenuate OSCC invasion through the manipulation of both local and systemic inflammatory mechanisms (**Figure 6**). Further, this response may be quantifiable and allow for disease prognostication and treatment in the setting of OPMDs or OSCCs.

## Data Availability Statement

The raw data supporting the conclusions of this article will be made available by the authors, without undue reservation.

## Ethics Statement

The studies involving human participants were reviewed and approved by University of Toronto Research Ethics Board (Protocol 38933). Written informed consent for the use of archived material was not required as per approved ethics protocol. The animal studies were reviewed and approved by University of Toronto Animal Care Committee (Protocol 20011940).

## Author Contributions

MM conceptualized the research. JC, RM, and AA prepared materials, collected, analyzed and interpreted the data. JC prepared the manuscript. MM supervised RM and MG supervised JC. Editing of the manuscript was performed by JC, AA, MG, and MM. All authors contributed to the article and approved the submitted version.

## Funding

JC is supported by the Canadian Institutes of Health Research (CIHR). RM is supported by the University of Toronto Faculty of Dentistry Summer Research Program. MM is supported by the Connaught New Researcher Award and a bridge grant (Priority Announcement for Oral Health) provided by the CIHR Institute of Musculoskeletal Health and Arthritis (PJ7-169674).

## Conflict of Interest

The authors declare that the research was conducted in the absence of any commercial or financial relationships that could be construed as a potential conflict of interest.

## Publisher’s Note

All claims expressed in this article are solely those of the authors and do not necessarily represent those of their affiliated organizations, or those of the publisher, the editors and the reviewers. Any product that may be evaluated in this article, or claim that may be made by its manufacturer, is not guaranteed or endorsed by the publisher.

## Supplementary Material

Supplementary Figure 1 4-NQO-induced carcinogenesis model characterization. **(A)** Representative oral photographs (left column) and longitudinal H&E low-power (middle column, 10X) and high-power (right column, 20X or 60X) photomicrographs of mouse oral tongue specimens. **(B)** Weight change of control (n=4) and 4-NQO treated (n=19) animals over the course of 4-NQO treatment and post-4-NQO observation. **(C)** Distribution of histologic diagnoses between 4-NQO-treated animals (n=19). *p < 0.01; **p < 0.0001; EH, Epithelial hyperplasia; LGD, Low-grade dysplasia (mild dysplasia); HGD, High-grade dysplasia (moderate and severe dysplasia); CIS, Carcinoma *in situ*; SCC, Squamous cell carcinoma; H&E, Hematoxylin and eosin.

Supplementary Figure 2 Cytokine Expression. Comparisons of cytokine concentration (pg/L) between post-4-NQO, interim and sacrifice time points for **(A)** control, **(B)** 4-NQO/isotype antibody treated and **(C)** 4-NQO/anti-TNFα antibody treated groups. MFI, Mean florescence intensity; 4-NQO, 4-Nitroquinoline 1-oxide; SEM, Standard error of the mean; GM-CSF, Granulocyte-macrophage colony-stimulating factor; G-CSF, Granulocyte colony-stimulating factor; INF, Interferon; IL, Interleukin; LIF, Leukemia inhibitory factor; LIX, Lipopolysaccharide-induced CXC chemokine; IP, Interferon γ-induced protein; KC, Keratinocytes-derived chemokine; MCP, Monocyte chemoattractant protein; MIP, Macrophage inflammatory protein; M-CSF, Macrophage-colony stimulating factor; MIG, Monokine induced by interferon-γ; RANTES, Regulated on activation, normal T cell expressed and secreted; VEGF, Vascular endothelial growth factor; TNFα, Tumor necrosis factor α.
