# Supplementary figures and images for: TNFα Signaling Is Increased in Progressing Oral Potentially Malignant Disorders and Regulates Malignant Transformation in an Oral Carcinogenesis Model
Source: Front Oncol. 2021 Sep 28;11:741013. doi: 10.3389/fonc.2021.741013 (PMC8507421; doi:10.3389/fonc.2021.741013)

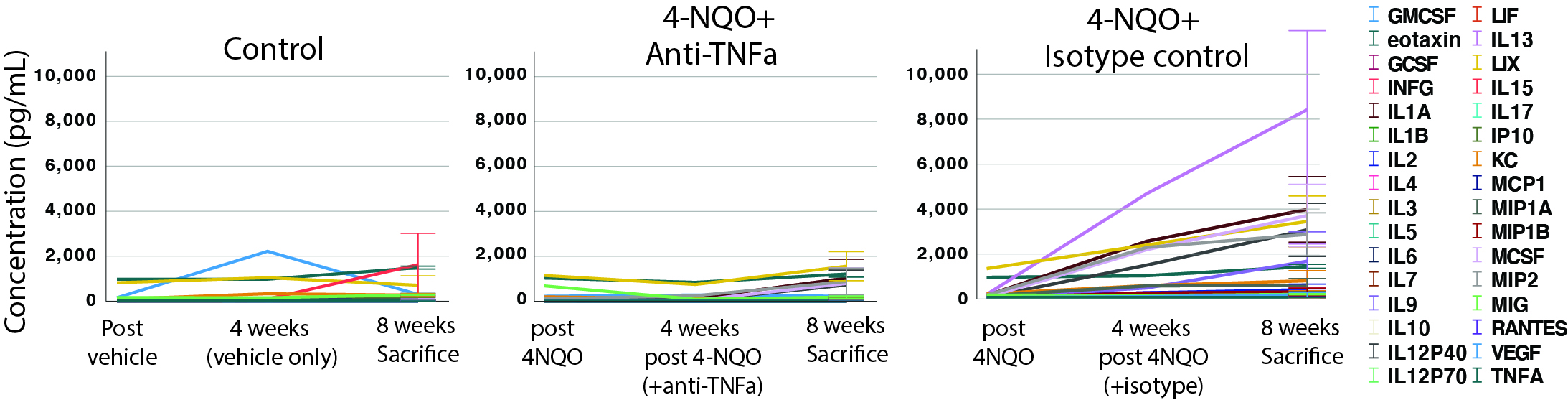

Supplement: Supplementary Figure 1 — 4-NQO-induced carcinogenesis model characterization. (A) Representative oral photographs (left column) and longitudinal H&E low-power (middle column, 10X) and high-power (right column, 20X or 60X) photomicrographs of mouse oral tongue specimens. (B) Weight change of control (n=4) and 4-NQO treated (n=19) animals over the course of 4-NQO treatment and post-4-NQO observation. (C) Distribution of histologic diagnoses between 4-NQO-treated animals (n=19). *p < 0.01; **p < 0.0001; EH, Epithelial hyperplasia; LGD, Low-grade dysplasia (mild dysplasia); HGD, High-grade dysplasia (moderate and severe dysplasia); CIS, Carcinoma in situ; SCC, Squamous cell carcinoma; H&E, Hematoxylin and eosin. [file Image_1.jpg]

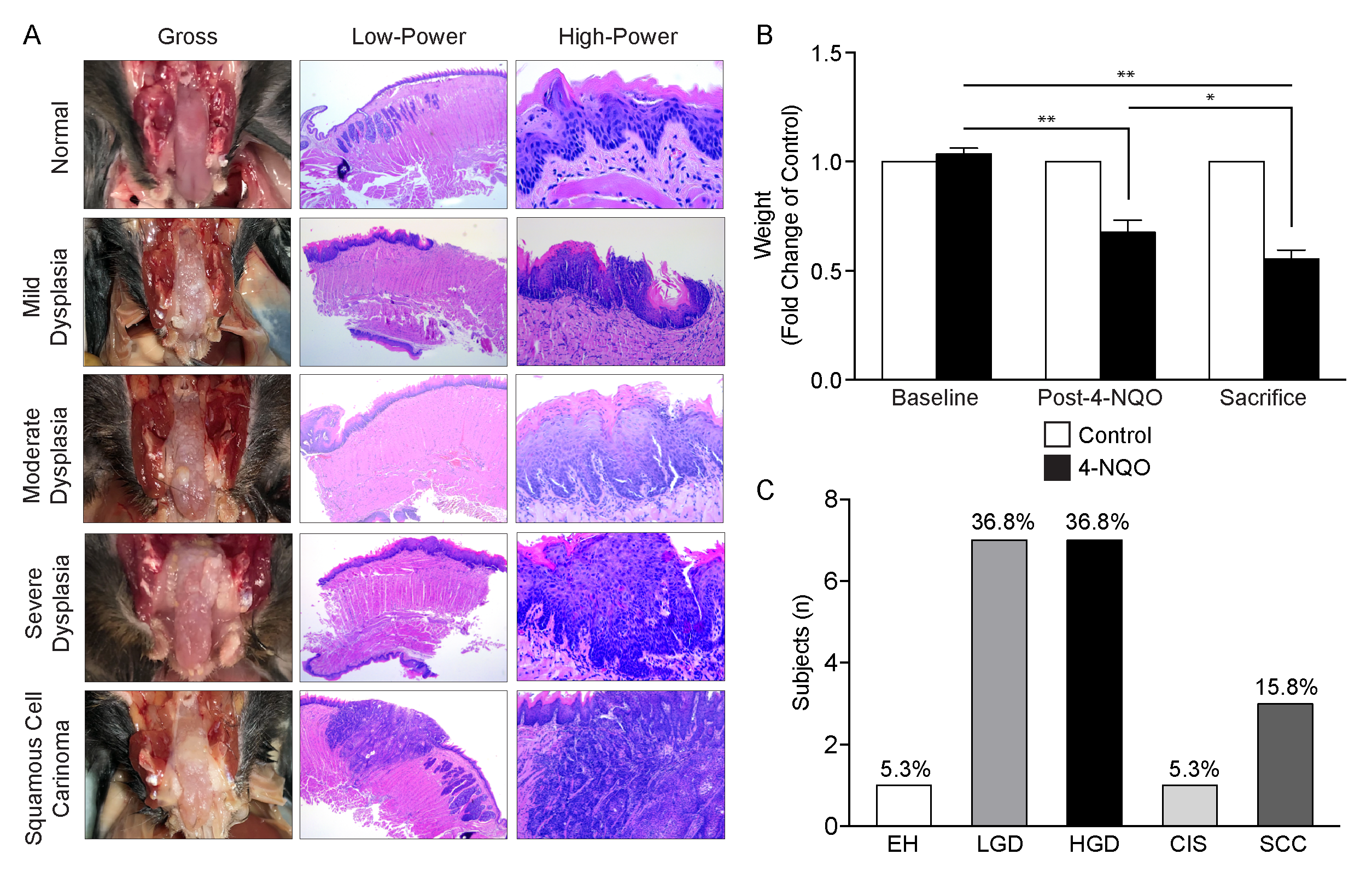

Supplement: Supplementary Figure 2 — Cytokine Expression. Comparisons of cytokine concentration (pg/L) between post-4-NQO, interim and sacrifice time points for (A) control, (B) 4-NQO/isotype antibody treated and (C) 4-NQO/anti-TNFα antibody treated groups. MFI, Mean florescence intensity; 4-NQO, 4-Nitroquinoline 1-oxide; SEM, Standard error of the mean; GM-CSF, Granulocyte-macrophage colony-stimulating factor; G-CSF, Granulocyte colony-stimulating factor; INF, Interferon; IL, Interleukin; LIF, Leukemia inhibitory factor; LIX, Lipopolysaccharide-induced CXC chemokine; IP, Interferon γ-induced protein; KC, Keratinocytes-derived chemokine; MCP, Monocyte chemoattractant protein; MIP, Macrophage inflammatory protein; M-CSF, Macrophage-colony stimulating factor; MIG, Monokine induced by interferon-γ; RANTES, Regulated on activation, normal T cell expressed and secreted; VEGF, Vascular endothelial growth factor; TNFα, Tumor necrosis factor α. [file Image_2.tiff]
