# Supplementary material for: TNFα Signaling Is Increased in Progressing Oral Potentially Malignant Disorders and Regulates Malignant Transformation in an Oral Carcinogenesis Model
Source: Front Oncol. 2021 Sep 28;11:741013. doi: 10.3389/fonc.2021.741013 (PMC8507421; doi:10.3389/fonc.2021.741013)
Supplement: Supplementary file 3 [file Table_1.docx]

|  | **Histopathologic Diagnosis** | **Sex** | **Age at Diagnosis**  **(Years)** | **Mean Age**  **(Years ± SD)** |
| --- | --- | --- | --- | --- |
| **Progressing cohort** | Severe Epithelial Dysplasia | F | 68 | 69.5 ± 11.9 |
|  |  | F | 60 |  |
|  | Moderate Epithelial Dysplasia | F | 74 |  |
|  |  | F | 49 |  |
|  |  | M | 66 |  |
|  | Mild Epithelial Dysplasia | F | 70 |  |
|  |  | M | 93 |  |
|  |  | F | 61 |  |
|  | Hyperkeratosis | M | 78 |  |
|  |  | F | 76 |  |
| **Control (non-progressing)** | Severe Epithelial Dysplasia | M | 84 | 60.2 ± 14.6 |
|  |  | M | 62 |  |
|  | Moderate Epithelial Dysplasia | F | Data Unavailable |  |
|  |  | M | 40 |  |
|  | Mild Epithelial Dysplasia | M | 54 |  |
|  |  | F | 79 |  |
|  |  | F | 67 |  |
|  | Hyperkeratosis | M | 47 |  |
|  |  | F | 59 |  |
|  |  | M | 50 |  |

**Supplemental Table 1: Patient demographics and associated histopathologic diagnoses.** Specimens were grouped for analysis as follows – Group 1: Hyperkeratosis; Group 2: Low-grade dysplasia: Mild epithelial dysplasia; Group 3: High-grade dysplasia (Moderate and severe epithelial dysplasia).
